# Development and Metrological Characterization of Low-Cost Wearable Pulse Oximeter

**DOI:** 10.3390/bioengineering12030314

**Published:** 2025-03-19

**Authors:** Andrea Cataldo, Enrico Cataldo, Antonio Masciullo, Raissa Schiavoni

**Affiliations:** Department of Engineering for Innovation, University of Salento, 73100 Lecce, Italy; enrico.cataldo@studenti.unisalento.it (E.C.); antonio.masciullo@unisalento.it (A.M.); raissa.schiavoni@unisalento.it (R.S.)

**Keywords:** pulse oximeter, arterial oxygen saturation *SpO*_2_, heart rate (HR) monitoring, low-cost medical devices, motion artifact reduction, signal processing

## Abstract

Pulse oximetry is essential for monitoring arterial oxygen saturation ($SpO_{2}$) and heart rate (HR) in various medical scenarios. However, the traditional pulse oximeters face challenges related to high costs, motion artifacts, and susceptibility to ambient light interference. This work presents a low-cost experimental pulse oximeter prototype designed to address these limitations through design advancements. The device incorporates a 3D-printed finger support to minimize motion artifacts and excessive capillary pressure, along with an elastic element to enhance stability. Unlike conventional transmission-based oximetry, the prototype employs a reflectance-based measurement approach, improving versatility and enabling reliable readings even in cases of poor peripheral perfusion. Additionally, the integration of light-shielding materials mitigates the effects of ambient illumination, ensuring accurate operation in challenging environments such as surgical settings. Metrological characterization demonstrates that the prototype achieves accuracy comparable to that of the commercial GIMA Oxy-50 pulse oximeter while maintaining a production cost at approximately one-tenth of the commercial alternatives. This study highlights the potential of the prototype to deliver affordable and reliable pulse oximetry for different applications.

## 1. Introduction

In recent years, advancements in wearable sensing technology and biomedical monitoring devices [1] have revolutionized the early diagnosis and continuous tracking of critical physiological parameters [2]. The evolution of low-cost technologies has enabled the development of increasingly sophisticated sensors capable of providing real-time, accurate measurements with applications in numerous medical fields. Among these, particular attention has been devoted to non-invasive diagnostics for cardiovascular [3,4], respiratory [5,6], and metabolic disorders, as well as the monitoring of neurodegenerative diseases [7] and cancer [8,9]. The integration of advanced signal processing techniques with wearable devices has significantly improved measurement reliability, making applications feasible in both clinical and home settings. Within this landscape, pulse oximetry [10] has emerged as one of the most widely used technologies for assessing respiratory and cardiovascular function. In clinical practice, the monitoring of the following six vital signs is fundamental for assessing an individual’s health status [11]: body temperature, heart rate, respiratory rate, blood pressure, cardiac index, and arterial oxygen saturation $SpO_{2}$ [12]. Among these, $SpO_{2}$ plays a crucial role in evaluating the body’s capacity to deliver adequate oxygen to organs and tissues (oxygenation). Defined as the ratio of oxygenated hemoglobin $\left(HbO_{2}\right)$ to the total amount of hemoglobin (both $\left(HbO_{2}\right)$ and deoxygenated (Hb) in arterial blood, $SpO_{2}$ is a critical parameter in a wide range of scenarios. These include preliminary screenings [13], emergency evaluations [14] (triage), surgical procedures, and postoperative monitoring [15], particularly in cases involving general anesthesia [16].

Despite its critical role in healthcare, pulse oximetry still faces different limitations. In particular, its widespread adoption is hindered by high costs, especially for CO-oximeters [17], susceptibility, and reduced reliability in specific conditions, such as low perfusion [18] or external interferences. Several low-cost pulse oximetry solutions have been proposed to address these issues, leveraging different sensor placements, signal processing techniques, and hardware optimizations [19,20]. However, the existing alternatives often show drawbacks. For instance, the low-cost smartphone-based pulse oximeter developed by Petersen et al. [21] relies on standard clinical sensors connected to a smartphone, reducing portability and requiring additional components for signal acquisition. Moreover, the traditional pulse oximeters may struggle in conditions of low peripheral perfusion, where reduced blood flow affects light absorption and compromises $SpO_{2}$ accuracy [21]. The device proposed by Adiputra et al. [22] adopts a clip-on sensor which can be highly sensitive to motion artifacts, leading to unstable readings. Furthermore, the pressure exerted by the clip may influence blood perfusion in the measurement site, introducing additional variability in the recorded signals. On the other hand, the low-cost pulse oximeter proposed by Agrawal et al. [23] prioritizes low power consumption but appears complex, requiring multiple interconnected components, which may limit its portability and practicality for real-world applications. Similar problem occurs in the study by Zaltum et al. [24]. Furthermore, the systems presented by Ali et al. [25], Rodrigues et al. [26], Naeem et al. [27], and Ganesh et al. [28] all lack adequate shielding against ambient light. In each case, the sensor remains fully exposed, increasing susceptibility to external optical interference. Moreover, the absence of proper finger support in these designs may lead to motion artifacts. To address these limitations, this study introduces a low-cost experimental pulse oximeter prototype designed to mitigate these challenges while maintaining affordability and usability. The proposed prototype introduces significant design advancements that enhance its performance in real-world applications, addressing the limitations commonly found in traditional pulse oximeters.

It is worth noting that pulse oximetry can be performed using two different measurement approaches—transmission-based and reflectance-based methods. The transmission method requires a sensor to be positioned on thin body parts, such as the fingertip or earlobe, allowing light to pass through the tissue before being detected on the opposite side [29]. This approach generally provides a strong signal and is commonly used in clinical pulse oximeters. However, it is less effective in patients with poor peripheral circulation, cold extremities, or thick tissue structures, where reduced light transmission may compromise measurement accuracy [30,31]. In contrast, the reflectance method, which is adopted in the proposed prototype, detects backscattered light from the underlying tissue, offering several advantages. Firstly, it allows for greater flexibility in sensor placement, making it suitable for alternative sites such as the forehead, wrist, or the back of the hand, where transmission-based sensors cannot be used. This characteristic is particularly beneficial in wearable applications and non-invasive continuous monitoring [32]. Additionally, the absence of clip-based pressure distribution reduces discomfort and allows for better adaptation to different anatomical conditions. Despite these advantages, reflectance-based sensors are generally more sensitive to external disturbances, including motion artifacts and variations in ambient light. To address these challenges, the proposed prototype integrates a custom-designed 3D-printed support with a glove-like elastic retention system. In particular, the custom-designed 3D-printed finger support effectively reduces motion artifacts while minimizing excess pressure on capillaries, which is one of the main drawbacks of conventional clip-based devices. This improvement ensures more accurate and consistent readings, even during patient movement. To further enhance stability, an adaptable elastic element securely holds the sensor in place, mitigating displacement-related inaccuracies. This solution minimizes the relative movement between the sensor and the skin while ensuring uniform pressure distribution over the finger. Unlike the traditional clip-based designs, where spring-loaded mechanisms exert localized pressure and may introduce measurement inconsistencies due to lateral displacement, the proposed design provides better mechanical stability. Furthermore, the optically shielding material is incorporated to significantly reduce interference from ambient light [33]. Moreover, the prototype overcomes a critical issue associated with the standard oximeters—measurement interference from synthetic nails or colored nail polish [34]. By leveraging reflectance-based technology, the device maintains high measurement accuracy despite these common obstacles. In addition, the integration of a protective glove made from optically shielding material significantly improves resistance to ambient light interference [33]. This feature is particularly beneficial in surgical and high-intensity lighting environments, where external illumination—such as fluorescent or xenon arc surgical lights [35]—often disrupts readings in conventional systems. Techniques such as Exponential Moving Average (EMA) filtering and sliding window-based Fast Fourier Transform (FFT) are employed to enhance noise reduction, preserve physiological signal components, and achieve high-resolution spectral analysis for accurate heart rate estimation. To rigorously validate the system’s performance, a comprehensive metrological characterization was conducted, directly comparing the experimental device against a well-established commercial reference, the GIMA Oxy-50 pulse oximeter. The analysis focuses on statistical metrics and demonstrates that the experimental device delivers comparable accuracy in measuring $SpO_{2}$ and heart rate. Importantly, the materials and fabrication techniques used in the prototype allow for a drastic reduction in production costs.

This paper is structured as follows: Section 2 provides the theoretical background, explaining the principles of pulse oximetry and its limitations. Section 3 describes the system design, including hardware improvements and signal processing techniques. Section 4 presents the metrological characterization and the experimental results, demonstrating the prototype’s performance compared to a commercial device. Finally, Section 5 summarizes the findings and discusses the potential applications and future developments.

## 2. Background

Pulse oximetry is a non-invasive technique widely used to measure arterial oxygen saturation $SpO_{2}$ and heart rate (HR), two critical vital signs for assessing an individual’s health status [36]. Photoplethysmography relies on the core principle of spectroscopic analysis described by the Beer–Lambert Law [37]. This law explains that the concentration of a light-absorbing substance in a solution can be calculated based on the light transmitted through it. The calculation requires knowledge of the intensity of the incident light, the path length of the light through the solution, and the substance’s extinction coefficient at a specific wavelength. Analytically, this relationship can be expressed as follows:

(1) $$A_{total}=\sum_{i=1}^{n}E_{i}C_{i}L_{i}$$

where, for each *i*-th substance, $A_{total}$ is the total absorption at a given wavelength; $E_{i}$ is the extinction coefficient (absorbency); $C_{i}$ is the concentration and $L_{i}$ is the path length. In the context of pulse oximetry, hemoglobin (Hb) and oxyhemoglobin ($HbO_{2}$) [38] serve as the primary chromophores in arterial blood, absorbing light at different rates depending on their oxygenation states. Red (660 nm) and infrared (940 nm) wavelengths are typically employed, as their absorption properties vary distinctly between Hb and $HbO_{2}$. These differences allow for the quantification of $SpO_{2}$ using a “ratio of ratios” approach. Specifically, the normalized absorption ratio R is calculated as follows:

(2) $$R=\frac{\frac{AC_{RED}}{DC_{RED}}}{\frac{AC_{IR}}{DC_{IR}}}$$

where $AC_{RED}$ and $AC_{IR}$ represent the pulsatile components of the red (660 nm) and infrared (940 nm) light signals, respectively, caused by periodic changes in the arterial blood volume synchronized with the heartbeat. On the other hand, $DC_{RED}$ and $DC_{IR}$ correspond to the non-pulsatile components of the red and infrared signals, which account for the constant absorption by the static tissues, such as skin, bones, and venous blood. This ratio eliminates the dependence on variations in incident light intensity and focuses on the relative light absorption by arterial blood. Through a calibration curve, $SpO_{2}$ is derived using a linear interpolation [39], typically expressed as follows:

(3) $$SpO_{2}=104+17\cdot R$$

where 104 and 17 are calibration coefficients that depend on demographic factors such as age, sex, and ethnicity. The pulsatile nature of the arterial signal ensures the discrimination of $SpO_{2}$ from static absorptions, enhancing the accuracy of the measurements.

## 3. System Realization

The proposed pulse oximetry system was designed with a strong emphasis on cost-effectiveness, leveraging carefully selected components to achieve an optimal balance among performance, versatility, and affordability. At the core of the system is the MAX30102 sensor, implemented via the “HiLetgo MAX30102” breakout board, which integrates essential components such as pull-up resistors, decoupling capacitors, and LDO voltage regulators. This setup simplifies the hardware design, reduces costs, and minimizes system size. The MAX30102 features red and infrared LEDs, a photodiode, and an 18-bit sigma-delta ADC, ensuring accurate measurements even in challenging conditions with ambient light interference. Its energy-efficient design supports continuous operation at less than 1 mW, while built-in features such as ambient light cancellation and a temperature sensor enhance accuracy and reliability. Data acquisition and software development are managed using a Raspberry Pi Pico W, selected for its low cost, support for I2C communication, and compatibility with MicroPython and C/C++. Although part of its memory is reserved for system resources, the remaining capacity is sufficient to support the development and experimentation needs. By integrating these components, the system achieves a streamlined and cost-efficient design with competitive performance. Furthermore, to overcome the common limitations of traditional pulse oximeters, such as motion artifacts and sensor misalignment, the system incorporates hardware and software innovations. These advancements, detailed in the following subsections, demonstrate a comprehensive approach to enhancing accuracy and usability.

### 3.1. Hardware Design

**Figure 1:**
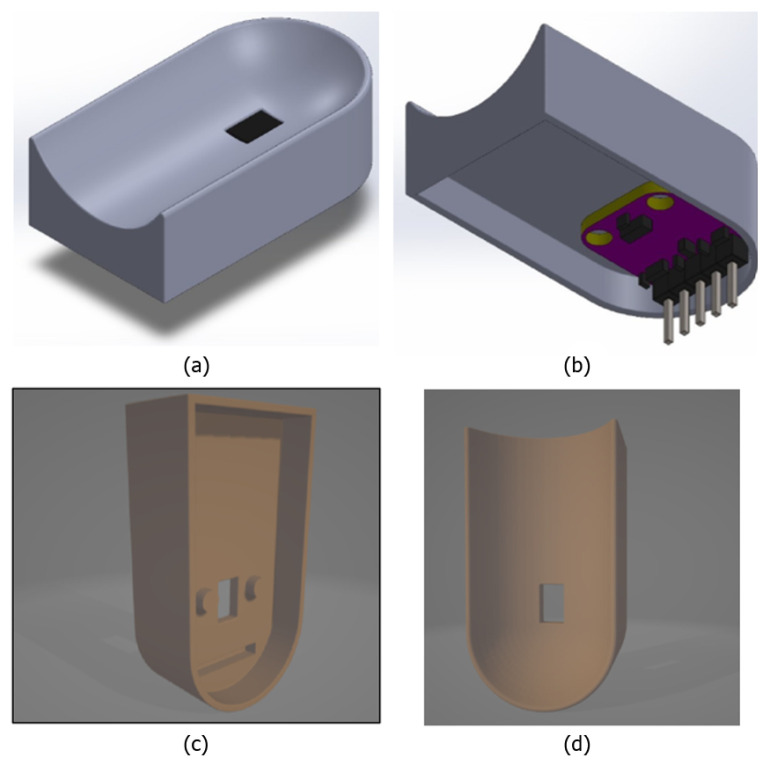
3D support. (**a**) External view of the ergonomic support; (**b**) internal view with sensor and electronic board; (**c**) structure with cylindrical supports for stability; (**d**) rear view with sensor opening.

**Figure 2:**
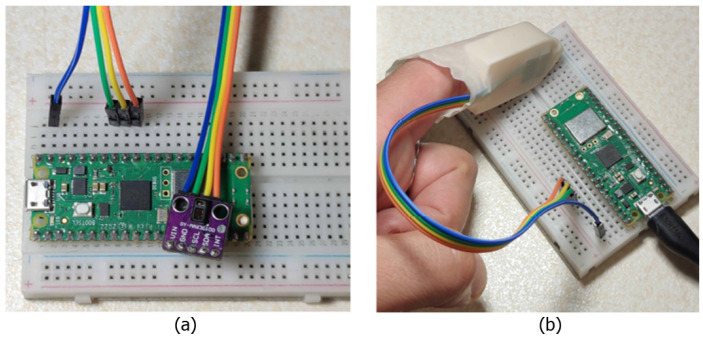
Complete system setup. (**a**) Sensor and microcontroller connected on a breadboard; (**b**) ergonomic support with mounted sensor, covered with a latex glove, and connected to the microcontroller.

Commercial pulse oximeters, such as clip-on or wearable models, present intrinsic limitations that can affect measurement accuracy. In clip-on devices, the optical sensor (comprising LEDs and a photodetector) is fixed using a spring that keeps the finger in place. However, this solution does not ensure a perfect alignment of the sensor with the skin and does not prevent micro-movements of the finger, which can introduce motion artifacts and compromise signal quality. Furthermore, these devices do not account for finger phalanx movements, which can alter capillary perfusion in the measurement area. Similarly, wearable devices, such as smartwatches, secure the sensor directly to the skin using a strap, offering greater comfort but failing to completely prevent misalignments or dynamic motion effects. To address these challenges, a three-dimensional support design for the pulse oximeter was developed, introducing significant improvements in hardware and mechanical stability. As shown in Figure 1, the support, conceived as a “thimble”, was designed to house the optical sensor in an optimal position and keep it firmly anchored. A central window ensures correct optical alignment between the LEDs, photodetector, and finger, minimizing misalignments. The sensor’s stability is further enhanced by two protruding cylinders on the back of the support, which fit into the eyelets of the sensor breakout board, drastically reducing micro-movements between the sensor and the skin. A latex glove is also introduced as a cost-effective and scalable solution to further stabilize the system. Acting as a flexible gripper, it securely holds the finger and rigid support in place while providing the necessary tension to inhibit phalanx articulation and ensure stability. This glove-based fixation mechanism allows for an automatic adaptation to different finger sizes, ensuring a firm yet comfortable fit without excessive pressure on the measurement site while mitigating ambient light interference. While the current prototype accommodates a broad range of finger sizes, future iterations will explore modular or adjustable elements, such as offering the device in three different sizes (small, medium, and large), to further enhance universality and user comfort. The calculated length of the rigid support inhibits joint movement between the distal and intermediate phalanges, ensuring stable capillary perfusion and reducing blood flow variations that could affect the signal. Unlike commercial devices, which rely on generic fixation systems, this solution specifically addresses finger dynamics, offering a more stable and accurate reading system. Figure 1 illustrates the design of the support and Figure 2 shows the complete system setup with the integration of the latex glove. It is worth noting that the proposed device was designed with cost-effectiveness in mind, utilizing low-cost components such as the Raspberry Pi Pico W (EUR 5), MAX30102 sensor (EUR 1), and a 3D-printed structural support (EUR 0.50). This results in an overall prototype cost that is significantly lower than those of the commercial pulse oximeters, such as the GIMA Oxy-50 (adopted in this study as reference device), which typically range from EUR 80 to EUR 100.

### 3.2. Data Acquisition and Processing

**Figure 3:**
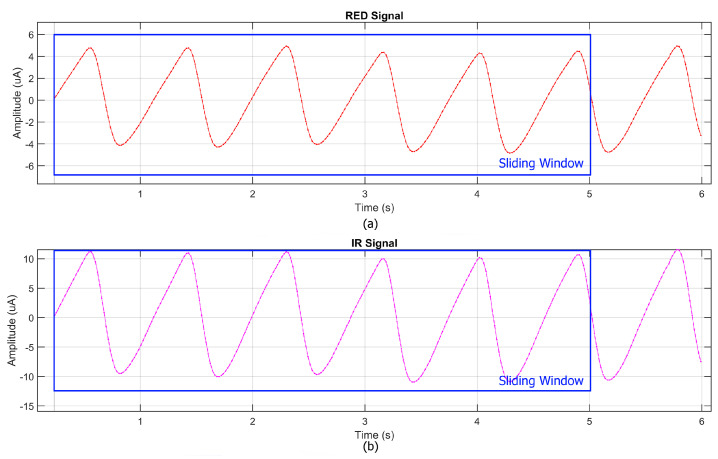
Example of a PPG signal with a sliding window technique applied, where a 5 s window shifts by 1 s to improve FFT spectral resolution. (**a**) RED signal, (**b**) IR signal.

The proposed system leverages MicroPython to implement an efficient and cost-effective workflow for data acquisition and processing. MicroPython was chosen for programming the Raspberry Pi Pico W due to its compatibility with the I2C protocol required by the MAX30102 sensor and its lightweight nature, making it ideal for resource-constrained microcontrollers. Before data acquisition, the sensor was configured via MicroPython, setting the LED intensity to its maximum and selecting an 18-bit resolution to optimize signal quality. Additionally, the decision to operate the sensor at 5 V instead of 3.3 V significantly improved the signal-to-noise ratio (SNR), addressing the noise issues observed in the preliminary tests. During acquisition, raw photoplethysmographic (PPG) signals were collected via I2C and stored in a CSV format on the microcontroller. Simultaneously, $SpO_{2}$ and heart rate (HR) measurements from the commercial pulse oximeter (GIMA Oxy-50) were recorded to enable direct comparison. After acquisition, the recorded data were transferred to MATLAB (version R2024b) for further processing and signal analysis. The processed signals from the prototype were then synchronized with the $SpO_{2}$ and HR values obtained from the commercial reference device. This synchronization allowed for a direct comparison between the two systems. The data processing pipeline included the use of the Exponential Moving Average (EMA), which was employed as both a low-pass and high-pass filter, ensuring an optimal balance between noise reduction and the preservation of physiological signal components. The heart rate (HR) estimation process generally begins with a spectral analysis and requires the resolution of frequency differences as small as 0.016 Hz, corresponding to a variation of just 1 beat per minute (bpm). Achieving this level of precision typically necessitates a 60 s stable signal; however, this would be impractical in physiological and real-time scenarios due to dynamic HR variations, motion artifacts, and other external factors. To address this limitation, a sliding window approach (Figure 3) was adopted, dividing the signal into 5 s overlapping segments, each shifted forward by 1 s. The FFT was applied to each segment, and results were aggregated to enhance frequency estimation while preserving temporal sensitivity. Zero-padding extended each segment to 4096 samples, refining spectral resolution without requiring longer recordings.

**Figure 4:**
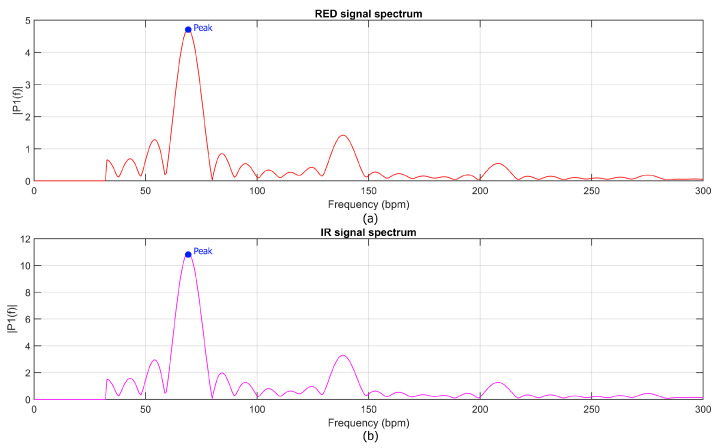
Power spectra of the (**a**) red (RED) and (**b**) infrared (IR) PPG signals showing the spectral peaks used to estimate heart rate in beats per minute (bpm).

Once the spectral representation was computed, the heart rate (HR) was estimated by identifying the prominent peaks in the spectra of both red and infrared signals, as shown in Figure 4. The HR was then calculated as the arithmetic mean of these two frequencies, ensuring robust estimation across the two channels. For oxygen saturation ($SpO_{2}$) estimation, the raw signal was processed using a similar approach to that employed for the heart rate (HR) analysis. The data, segmented into 1 s temporal windows, underwent an initial low-pass filtering using an EMA filter to reduce noise while preserving the dynamic range of the signal.

**Figure 5:**
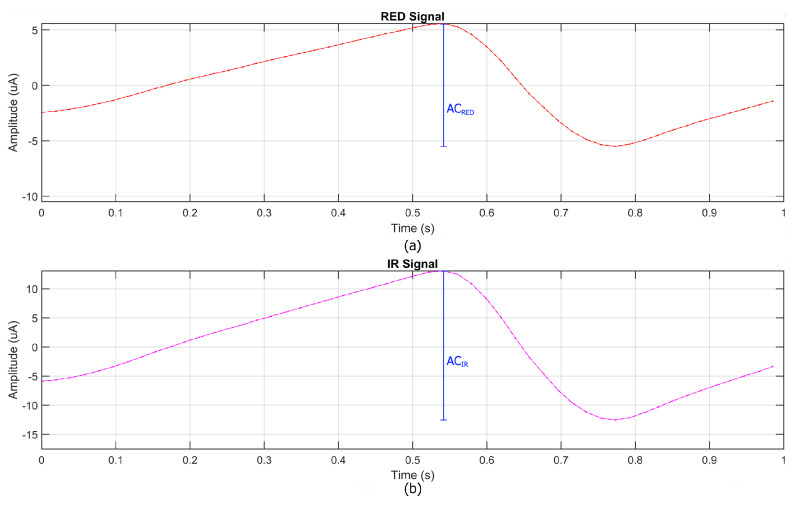
Illustration of the pulsatile AC component in the (**a**) RED and (**b**) IR signals used in pulse oximetry. The AC amplitude represents the variation in light absorption due to arterial blood flow.

Subsequently, the signals were centered by removing their direct current (DC) components, calculated as the local minimum within each temporal window, ensuring that the signals were aligned to zero. From the processed signals, both the direct current (DC) and alternating current (AC) components were extracted for the RED and IR channels, as shown in Figure 5. The DC components represent the average signal level over the temporal window, while the AC components correspond to the pulsatile nature of arterial blood flow. Using these components, the ratio R was calculated according to Equation (2). $SpO_{2}$ was then estimated using Equation (3). This approach ensures accurate $SpO_{2}$ estimation by effectively separating the pulsatile (AC) and baseline (DC) components, which are essential for deriving oxygen saturation. Additionally, while further high-pass filtering can be employed to refine the signal, its impact on the signal’s dynamic range is carefully evaluated to preserve the original signal integrity.

## 4. Metrological Assessment and Experimental Methodology

To validate the performance of the developed device, a comprehensive comparison was conducted against a professional-grade pulse oximeter (GIMA Oxy-50). The study involved the simultaneous acquisition of $SpO_{2}$ and heart rate (HR) data across 10 subjects under different physiological conditions. Since physiological parameters like $SpO_{2}$ and HR are inherently subject-dependent and continuously fluctuating, establishing an absolute ground truth is challenging in real-world conditions. For this reason, commercial pulse oximeters, such as the GIMA Oxy-50, are commonly adopted as reference devices, as they undergo regulatory testing and are widely used in clinical practice. While not infallible, they provide a practical and validated benchmark for performance comparison. The following subsections describe the accuracy metrics adopted and the experimental protocol in detail.

### 4.1. Metrics for Accuracy Assessment

To quantitatively evaluate the accuracy of the developed pulse oximeter, a comparison was performed against a commercial-grade reference device, the GIMA Oxy-50. The assessment was conducted using the following standard statistical metrics commonly employed in biomedical signal validation:

- Bias, representing the average difference between the prototype and the reference device as a measure of accuracy, defined as follows:
  (4) $$Bias=\frac{1}{n}\sum_{i=1}^{n}\left(x_{i}-y_{i}\right)$$
- Standard Deviation (*STD*), quantifying the dispersion of the differences, defined as follows:
  (5) $$STD=\sqrt{\frac{1}{n-1}\sum_{i=1}^{n}{\left(\left(x_{i}-y_{i}\right)-Bias\right)}^{2}}$$

where $x_{i}$ is the measurement from the prototype device, $y_{i}$ is measurement from the reference device (GIMA Oxy-50), and n is the total number of measurement pairs. The total uncertainty, $U_{comb}$, was calculated as the root sum of the squares of the bias and standard deviation, as follows:

(6) $$U_{comb}=\sqrt{Bias^{2}+STD^{2}}$$

In addition, the Bland–Altman plot was adopted since it is a widely used statistical method for evaluating the agreement between two measurement systems. This method provides a visual representation of the differences between the values measured by the experimental device and the reference device, allowing for the identification of potential systematic bias and the range of variability.

### 4.2. Experimental Protocol and Testing Conditions

To validate the performance of the developed device under real-world physiological conditions, an experimental study on 10 healthy adult participants (5 males and 5 females, aged between 20 and 40 years) was conducted. The protocol was designed to capture the $SpO_{2}$ and heart rate (HR) measurements across three distinct physiological states, simulating different clinical and daily life scenarios, such as the following:

- Resting State (Baseline Condition);
- Post-Light Physical Activity (Stretching Routine);
- Post-Intense Physical Activity (Running in Place at Controlled Cadence).

In the resting state condition, each participant was asked to sit in a comfortable indoor environment at a controlled room temperature (22–24 °C) to minimize external thermal influences. Before starting the data collection, the subjects remained seated for at least 10 min to ensure cardiovascular stabilization. To minimize involuntary motion artifacts, their hands were placed on a stable surface during the measurement phase. Subsequently, to evaluate the performance under mild physiological variations, the participants engaged in a 5 min guided stretching routine consisting of the following: (i) arm and shoulder stretches (15 s per side); (ii) torso rotations (20 repetitions); and (iii) quadriceps and hamstring stretches (15 s per leg). Immediately after completion, the subjects remained seated, and the $SpO_{2}$ and HR values were recorded within 150 s, allowing for natural post-exercise tremors to be observed. Finally, to simulate high-motion scenarios, the participants performed a high-knee running-in-place exercise synchronized with a metronome set to 180 beats per minute (bpm). The exercise lasted for 60 s, ensuring a consistent cadence across all the participants. Upon completion of the exercise, the subjects were guided back to a seated position, and measurements were taken within 150 s, capturing post-exercise hand tremors and increased respiratory movement.

## 5. Experimental Results

**Figure 6:**
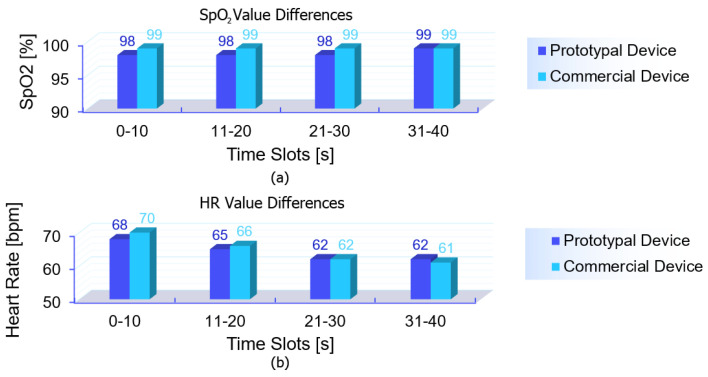
Overview of measurements comparing the experimental device and the GIMA Oxy-50 for $SpO_{2}$ and heart rate. (**a**) $SpO_{2}$ values across 10 s time slots; (**b**) heart rate values across 10 s time slots.

**Figure 7:**
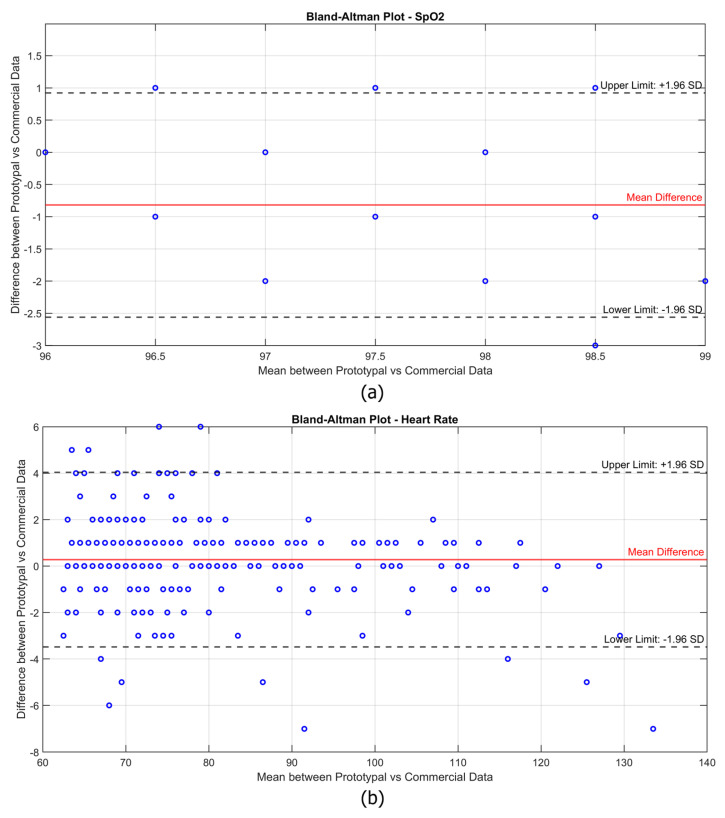
Bland–Altman plot for (**a**) $SpO_{2}$ and (**b**) heart rate (HR) measurements comparing the prototype with the commercial reference (GIMA Oxy-50). The red line represents the mean difference (bias), while the dashed lines indicate the limits of agreement (±1.96 SD).

Before delving into the detailed metrological evaluation, a simplified representation of the collected data is provided to offer an overview of the differences observed between the devices. A moving average smoothing algorithm was applied, averaging the last five recorded values over time. These smoothed values were further aggregated every 10 s, generating paired data points that allow for a clearer comparison with the commercial device. This step was implemented solely to enhance data visualization, reducing noise and highlighting trends, as shown in Figure 6. The comparison shows that the experimental device closely tracked the reference device across the different conditions, with slight variations in both $SpO_{2}$ and heart rate (HR) values. Furthermore, the Bland–Altman plots in Figure 7 illustrate the agreement between the experimental prototype and the commercial reference device.

Each subject was measured for 150 s in each condition, with data recorded at a frequency of one sample per second. However, rather than analyzing each individual second, the data were aggregated into time slots of 10 s, meaning that each condition resulted in 15 total measurements per subject. The first measurement of each sequence was discarded to avoid transient effects, leaving 14 valid data points per condition per subject. This approach helped reduce momentary fluctuations while still maintaining a sufficiently high temporal resolution for analysis. As a result, rather than having one data point per condition per subject, the Bland–Altman plots contain a considerable number of aggregated measurements, reflecting the continuous acquisition process while ensuring statistical robustness. It is worth noting that for $SpO_{2}$, the data points appear more concentrated due to the overlapping of multiple points. This is because the measurements were performed on healthy subjects, whose oxygen saturation levels were typically stable in the 97–99% range. Consequently, many values coincide, resulting in a visually lower density of points in the plot despite the high number of data points recorded. A similar effect is also present in the HR data, although to a lesser extent, as some data points still overlap, reducing their apparent density in the plot. However, the overall data distribution for HR is more spread out due to natural variations, particularly under different physical activity conditions. Unlike $SpO_{2}$, which remains relatively stable in healthy individuals, heart rate varies significantly between the resting and post-exercise states. This wider variability leads to a more scattered distribution of points in the plot, although all the measurements remain within the limits of agreement. Overall, the Bland–Altman analysis confirms that the experimental device provides measurements comparable to the commercial reference, with the differences remaining within the acceptable limits for clinical applications. In the case of $SpO_{2}$, the mean difference between the two devices is slightly negative, suggesting a minor underestimation of blood oxygen saturation by the prototype compared to the commercial device. However, the differences remain within the upper and lower limits of agreement, with no significant systematic bias affecting measurement reliability. The limited variability in the $SpO_{2}$ values further reinforces the robustness of the prototype, as no extreme deviations are observed. For heart rate (HR), the mean difference is close to zero, indicating good overall agreement with the reference device. In addition, the vast majority of data points remain well within the limits of agreement, confirming that the experimental device maintains reliable performance even under dynamic conditions.

Finally, to quantitatively assess the accuracy of the developed device, the key statistical metrics were computed, including bias, standard deviation (*STD*), and combined uncertainty ($U_{c}omb$). The results indicate that the prototype achieves a total accuracy of ±0.95% for the $SpO_{2}$ measurements and ±1.69 bpm for heart rate (HR), demonstrating a performance comparable to that of the commercial reference device (GIMA Oxy-50). Notably, the experimental device outperforms the reference device in $SpO_{2}$ accuracy, which is specified at ±2% for GIMA Oxy-50. For the HR, the achieved accuracy of ±1.69 bpm remains well within the commercial benchmark of ±2 bpm or ±2%, further validating the reliability of the prototype across different physiological conditions. A comparative summary of the performance metrics is presented in Table 1, highlighting the strong agreement between the prototype and the reference device.

## 6. Discussion and Contextualization

Based on the experimental results presented in the previous section, in addition to the comparison with GIMA Oxy-50, this discussion aims to contextualize the performance of the developed prototype within the broader landscape of low-cost pulse oximetry solutions. Table 2 presents a comparative analysis of the developed prototype with existing commercial low-cost pulse oximeters, along with those reported in the literature. The comparison considers the measurement method and its accuracy. Table 2 highlights how the proposed device achieves a lower error margin for $SpO_{2}$ measurements compared to the commercial and research-based pulse oximeters while maintaining a significantly reduced production cost.

In addition to the good results in terms of measurement accuracy, unlike several other low-cost solutions, the developed prototype integrates mechanical stabilization features and ambient light shielding, enhancing signal reliability. The reflectance-based measurement approach may also offer greater versatility by enabling placement beyond the fingertip, where transmission-based sensors can encounter limitations, and ensuring measurement robustness even in the presence of synthetic nails or colored nail polish. While further testing is necessary to fully assess its robustness across diverse conditions and populations, the design choices adopted in this study aim to mitigate some common challenges in pulse oximetry. In particular, the 3D-printed support and latex membrane were introduced to enhance mechanical stability, potentially reducing motion artifacts and preventing excessive capillary pressure, which can affect perfusion in traditional clip-based designs. Additionally, the possibility of selecting optically shielding materials for the latex membrane further reduces ambient light interference, improving reliability in environments with strong artificial lighting, such as surgical settings. Finally, the use of readily available, low-cost components may facilitate future developments toward miniaturization and the integration with wearable platforms.

## 7. Conclusions and Future Works

In this study, a cost-effective pulse oximetry prototype was developed and experimentally validated on 10 subjects across three different conditions. The results confirmed the device’s reliability across varying physiological states, including scenarios prone to motion artifacts. The metrological characterization demonstrated comparable accuracy in the $SpO_{2}$ and HR measurements to those of a commercial pulse oximeter, as further supported by Bland–Altman analysis. The key design features include a 3D-printed support to reduce motion artifacts, a reflectance-based approach for greater versatility in site placement, and the mitigation of ambient light interference for improved robustness. Several areas remain for future investigation and improvement. First, expanding the validation to larger and more diverse populations will be crucial to further assess the device’s generalizability and clinical reliability. Additionally, integrating advanced signal processing algorithms, such as machine learning-based artifact removal, could enhance measurement accuracy under motion-intensive scenarios. Future developments will also focus on system miniaturization and packaging, aiming to integrate a fully enclosed and portable version of the device with embedded data processing capabilities. Moreover, while the initial cost estimations were based on component-level pricing, the future iterations will focus on optimizing design choices and production methods to maintain affordability while ensuring compliance with medical device standards. To achieve this, large-scale manufacturing will be considered to reduce production costs, and hardware design will be refined through the integration of a custom PCB. Additionally, to keep costs low, the device will not include a built-in display, relying instead on a smartphone-based application for real-time data visualization and device control. A miniaturized lithium battery will also be explored to enhance portability without significantly impacting affordability. Future iterations of the device could also explore the use of alternative sensor materials, such as organic photodetectors, to further improve sensitivity and energy efficiency. Finally, expanding the system to support real-time data transmission via wireless connectivity would enable remote monitoring applications, broadening its potential impact in telemedicine and home healthcare settings.

## Figures and Tables

**Table 1:** Comparison of performance metrics between the commercial device (GIMA OXY-50) and the experimental prototype for $SpO_{2}$ and heart rate measurements, highlighting accuracy, resolution, and measurement range.

		Commercial Device	Prototypal Device
**SpO_2_**	Measured range	0–100%	0–100%
	Accuracy	±2%	±0.95%
	Resolution	1%	1%
**Heart Rate**	Measured range	30–250 bpm	30–300 bpm
	Accuracy	±2 bpm or ±2% whichever is greater.	±1.69 bpm
	Resolution	1 bpm	1 bpm

**Table 2:** Comparison of proposed prototype with commercial and research-based pulse oximeters.

Device	Device Type	Measurement Method	SpO_2_ Accuracy (%)	HR Accuracy (bpm)
**Proposed Device**	Prototype	Reflectance	±0.95	±1.69
**Banik et al. [20]**	Prototype	Reflectance	±1.04	±2.08
**Adiputra et al. [22]**	Prototype	Transmittance	±1.50	±2.80
**Peterson et al. [21]**	Prototype	Transmittance	±1.65	Not reported
**Naeem et al. [27]**	Prototype	Reflectance	±1.46	±3.00
**Zaltum et al. [24]**	Prototype	Reflectance	±5.00	Not reported
**Contec CMS50D**	Commercial	Transmittance	±2.00	±2.00
**Nonin 8000R**	Commercial	Reflectance (Frontal)	±2.00	±3.00
**GIMA Oxy-50**	Commercial	Transmittance	±2.00	±2.00
**Masimo Radical-7**	Commercial	Transmittance	±2.00	±3.00

## Data Availability

The raw data supporting the conclusions of this article will be made available by the authors on request.

## Abbreviations

The following abbreviations are used in this manuscript:

$SpO_{2}$	Arterial Oxygen Saturation
EMA	Exponential Moving Average
FFT	Fast Fourier Transform
$HbO_{2}$	Oxygenated Hemoglobin
Hb	Hemoglobin
DC	Direct Current
AC	Alternating Current
SNR	Signal-to-Noise Ratio
ADC	Analog-to-Digital Converter
LDO	Low Dropout Regulator
I2C	Inter-Integrated Circuit
PPG	Photoplethysmography
bpm	Beats Per Minute
Ucomb	Combined Uncertainty

## References

[1] Ahmed I., Balestrieri E., Lamonaca F. (2021). IoMT-based biomedical measurement systems for healthcare monitoring: A review. Acta IMEKO.

[2] Jegan R., Nimi W.S. (2024). On the development of low power wearable devices for assessment of physiological vital parameters: A systematic review. J. Public Health.

[3] Bayoumy K., Gaber M., Elshafee A., Mhaimeed O., Dineen E.H., Marvel F.A., Martin S.S., Muse E.D., Turakhia M.P., Tarakji K.G. (2021). Smart wearable devices in cardiovascular care: Where we are and how to move forward. Nat. Rev. Cardiol..

[4] Hughes A., Shandhi M.M.H., Master H., Dunn J., Brittain E. (2023). Wearable Devices in Cardiovascular Medicine. Circ. Res..

[5] Hussain T., Ullah S., Fernández-García R., Gil I. (2023). Wearable Sensors for Respiration Monitoring: A Review. Sensors.

[6] Vitazkova D., Foltan E., Kosnacova H., Micjan M., Donoval M., Kuzma A., Kopani M., Vavrinksy E. (2024). Advances in Respiratory Monitoring: A Comprehensive Review of Wearable and Remote Technologies. Biosensors.

[7] Cataldo A., Criscuolo S., De Benedetto E., Mascilupo A., Pesola M., Picone J., Schiavoni R. (2024). EEG complexity-based algorithm using Multiscale Fuzzy Entropy: Towards a detection of Alzheimer’s disease. Meas. J. Int. Meas. Confed..

[8] Cataldo A., Cino L., Distante C., Maietta G., Masciullo A., Mazzeo P.L., Schiavoni R. (2024). Integrating microwave reflectometry and deep learning imaging for in-vivo skin cancer diagnostics. Meas. J. Int. Meas. Confed..

[9] Pulumati A., Pulumati A., Dwarakanath B.S., Verma A., Papineni R.V.L. (2023). Technological advancements in cancer diagnostics: Improvements and limitations. Cancer Rep..

[10] Wukitsch M.W., Petterson M.T., Tobler D.R., Pologe J.A. (1988). Pulse oximetry: Analysis of theory, technology, and practice. J. Clin. Monit..

[11] Lockwood C.R.B.G.M., Conroy-Hiller T.R.B.D.G., Page T.R.B.H.G.M. (2004). Vital signs. JBI Libr. Syst. Rev..

[12] Shelley K.H. (2007). Photoplethysmography: Beyond the Calculation of Arterial Oxygen Saturation and Heart Rate. Anesth. Analg..

[13] Plana M.N., Zamora J., Suresh G., Fernandez-Pineda L., Thangaratinam S., Ewer A.K. (2018). Pulse oximetry screening for critical congenital heart defects. Cochrane Database Syst. Rev..

[14] Lee W.W., Mayberry K., Crapo R., Jensen R.L. (2000). The accuracy of pulse oximetry in the emergency department. Am. J. Emerg. Med..

[15] Kyriacou P.A., Powell S., Langford R.M., Jones D.P. (2002). Investigation of oesophageal photoplethysmographic signals and blood oxygen saturation measurements in cardiothoracic surgery patients. Physiol. Meas..

[16] Yamakage M., Namiki A., Tsuchida H., Iwasaki H. (1992). Changes in ventilatory pattern and arterial oxygen saturation during spinal anaesthesia in man. Acta Anaesthesiol. Scand..

[17] Papin M., Latour C., Leclère B., Javaudin F. (2023). Accuracy of pulse CO-oximetry to evaluate blood carboxyhemoglobin level: A systematic review and meta-analysis of diagnostic test accuracy studies. Eur. J. Emerg. Med..

[18] Hummler H.D., Engelmann A., Pohlandt F., Högel J., Franz A.R. (2006). Decreased accuracy of pulse oximetry measurements during low perfusion caused by sepsis: Is the perfusion index of any value?. Intensive Care Med..

[19] Garanin A.A., Kolsanov A.V., Shipunov I.D. (2024). Review of the world market for pulse oximeter medical devices. Biomed. Eng..

[20] Banik P.P., Hossain S., Kwon T.H., Kim H., Kim K.D. (2020). Development of a Wearable Reflection-Type Pulse Oximeter System to Acquire Clean PPG Signals and Measure Pulse Rate and SpO_2_ with and without Finger Motion. Electronics.

[21] Petersen C.L., Chen T.P., Ansermino J.M., Dumont G.A. (2013). Design and Evaluation of a Low-Cost Smartphone Pulse Oximeter. Sensors.

[22] Adiputra R.R., Hadiyoso S., Hariyani Y.S. (2018). Internet of Things: Low Cost and Wearable SpO2 Device for Health Monitoring. Int. J. Electr. Comput. Eng. (IJECE).

[23] Agrawal N., Agrawal S., Kumar A., Kini M.R. Optimized Low Power Low Cost Pulse Oximeter for Remote Patient Monitoring. Proceedings of the 2013 Texas Instruments India Educators’ Conference (TIIEC).

[24] Zaltum M.A., Ahmad M.S., Joret A., Jamil M.M.A. (2021). Design and Development of a Portable Pulse Oximetry System. Int. J. Integr. Eng..

[25] Ali M.M., Haxha S., Alam M.M., Nwibor C., Sakel M. (2020). Design of Internet of Things (IoT) and Android Based Low Cost Health Monitoring Embedded System Wearable Sensor for Measuring SpO_2_, Heart Rate and Body Temperature Simultaneously. Wirel. Pers. Commun..

[26] Rodrigues E.M.G., Godina R., Cabrita C.M.P., Catalão J.P.S. (2017). Experimental low cost reflective type oximeter for wearable health systems. Biomed. Signal Process. Control..

[27] Naeem Z.H., Youseffi M., Sefat F., Khaghani S.A., Raja T.I., Patel A., Javid F., Jamil M.M.A., Wahab M.H.A. (2021). Design and Development of a Low Cost Pulse Oximeter. J. Phys. Conf. Ser..

[28] Ganesh K.V.S.S., Jeyanth S.P.S., Bevi A.R. (2022). IOT based portable heart rate and SpO2 pulse oximeter. HardwareX.

[29] Pälve H. (1992). Reflection and transmission pulse oximetry during compromised peripheral perfusion. J. Clin. Monit..

[30] Allen J. (2007). Photoplethysmography and its application in clinical physiological measurement. Physiol. Meas..

[31] Tamura T., Maeda Y., Sekine M., Yoshida M. (2014). Wearable Photoplethysmographic Sensors—Past and Present. Electronics.

[32] Lee J., Kim C. (2018). Advances in Photoplethysmography: A Review. IEEE Sens. J..

[33] Amar D., Neidzwski J., Wald A., Finck A.D. (1989). Fluorescent light interferes with pulse oximetry. J. Clin. Monit..

[34] Coté C.J., Goldstein E.A., Fuchsman W.H., Hoaglin D.C. (1988). The effect of nail polish on pulse oximetry. Anesth. Analg..

[35] Schulz E.B., Ham J.A. (2019). Light-emitting diode surgical light interference with pulse oximetry. Br. J. Anaesth..

[36] Tremper K.K. (1989). Pulse Oximetry. Chest.

[37] Mayerhöfer T.G., Mutschke H., Popp J. (2016). Employing Theories Far beyond Their Limits—The Case of the (Bouguer-) Beer–Lambert Law. ChemPhysChem.

[38] Giardina B., Messana I., Scatena R., Castagnola M. (1995). The Multiple Functions of Hemoglobin. Crit. Rev. Biochem. Mol. Biol..

[39] Antoniou P., Nestoros M., Polycarpou A.C. (2023). Calculation of Heartbeat Rate and SpO_2_ Parameters Using a Smartphone Camera: Analysis and Testing. Sensors.

